# Clinicopathological and prognostic significance of cyclooxygenase-2 expression in head and neck cancer: A meta-analysis

**DOI:** 10.18632/oncotarget.10059

**Published:** 2016-06-15

**Authors:** Bin Yang, Lin Jia, Qiaojuan Guo, Hui Ren, Yanping Hu, Tao Xie

**Affiliations:** ^1^ Department of Oncology, Hubei Cancer Hospital, Wuhan, Hubei 430079, China; ^2^ Department of Nephrology, The Central Hospital of Wuhan, Wuhan, Hubei 430014, China; ^3^ Department of Radiation Oncology, Fujian Provincial Cancer Hospital, Provincial Clinical College of Fujian Medical University, Fuzhou, Fujian 350000, China; ^4^ Department of Radiation Oncology, Hubei Cancer Hospital, Wuhan, Hubei 430079, China

**Keywords:** head and neck cancer, cyclooxygenase-2, prognosis, biomarker, meta-analysis

## Abstract

Several studies have assessed the clinicopathological and prognostic value of cyclooxygenase-2 (COX-2) expression in patients with head and neck cancer (HNC), but their results remain controversial. To address this issue, a meta-analysis was carried out. A total of 29 studies involving 2430 patients were subjected to final analysis. Our results indicated that COX-2 expression was not statistically associated with advanced tumor stage (OR, 1.23; 95% CI, 0.98–1.55) but correlated with high risk of lymph node metastasis (OR, 1.28; 95% CI, 1.03–1.60) and advanced TNM stage (OR, 1.33; 95% CI, 1.06–1.66). Moreover, COX-2 expression had significant effect on poor OS (HR, 1.93; 95% CI, 1.29–2.90), RFS (HR, 2.02; 95% CI, 1.00–4.08) and DFS (HR, 5.14; 95% CI, 2.84–9.31). The results of subgroup analyses revealed that COX-2 expression was related with high possibility of lymph node metastasis in oral cancer (OR, 1.49; 95% CI, 1.01–2.20) and advanced TNM stage in oral cancer (OR, 1.58; 95% CI, 1.05–2.37) and no site-specific HNC (OR, 1.64; 95% CI, 1.02–2.62). However, subgroup analyses only showed a tendency without statistically significant association between COX-2 expression and survival. Significant heterogeneity was not found when analyzing clinicopathological data, but it appeared when considering survival data. No publication bias was detected in this study. This meta-analysis suggested that COX-2 expression could act as a prognostic factor for patients with HNC.

## INTRODUCTION

Head and neck cancer (HNC) comprise a series of tumors arising in the lip, oral cavity, pharynx and larynx. It was reported that there were an estimated 387,100 new cases of HNC and 196,200 deaths in 2012 worldwide [1]. Despite the development in diagnosis and treatment, the survival of patients with HNC is still not significantly improved, with 5-year survival rate approximately 40%–50% [2, 3]. Therefore, it is necessary to explore strategies to screen out high risk patients and predict outcome of this disease. Nowadays, the most accepted prognostic factor for HNC is TNM classification system. However, the TNM system is not always effective in providing prognostic information for HNC. Several studies suggested that none of the TNM systems used or proposed could account for even 30% of the variation observed in the survival rates of HNC [4–6]. The possible interpretation may be that TNM system classifies the extent of disease chiefly on the basis of anatomic information and cannot reflect the biological heterogeneity of cancer. Thus, identifying molecular markers associated with the biological behavior of HNC may complement the TNM system in the prognostication of HNC.

It is now generally accepted that inflammatory microenvironment plays critical roles in tumor development [7]. The cyclooxygenase-2 (COX-2), one of the two isoforms of COX, is usually unexpressed in most normal tissues but rapidly induced by mitogenic and inflammatory stimuli [8]. Inflammation-induced COX-2 has been reported to participate in the development and survival of cancers [9–11]. Besides, COX-2 is widely expressed and profoundly linked to poor prognosis in a variety of malignant tumors [12, 13]. What's more, inhibition of COX-2 has a reversed effect on cancer progression [14–18]. Therefore, COX-2 might be a potential prognostic factor to predict the survival in patients with cancers.

Previous experimental studies have shown that COX-2 has an important role in the growth and metastasis of HNC by a variety of pathways [11, 19]. Moreover, the effect of COX-2 inhibitors has been analyzed clinically for patients with HNC and targeting COX-2 seems to be an effective way to control HNC [20, 21]. Thus, COX-2 may have a prognostic function in HNC. Numerous studies have examined the relationship between COX-2 expression and survival in patients with HNC [22–27]. However, the clinicopathological and prognostic role of COX-2 in HNC has yet to be confirmed. First, the sample sizes in these published studies are often small (see Table 1). Second, the existing studies are conflicting in their results. Some studies suggested that COX-2 expression was associated with poor prognosis in HNC [22–24], whereas other studies failed to demonstrate such correlation [25–27]. Therefore, it is still difficult to determine the prognostic value of COX-2 expression. Accordingly, we performed a meta-analysis to evaluate the clinicopathological and prognostic role of COX-2 expression in patients with HNC.

**Table 1:** Characteristics of studies included in this meta-analysis

First author	Tumor types	Patients	COX-2 assay	Cutoff level	Clinicopathological factors	Survival results
Byatnal (2015) [37]	OC	75	IHC	> 5%	N	None
Morita (2014) [38]	OC	40	IHC	> 5%	T, N, TNM	OS
Kono (2013) [22]	OC	60	IHC	> 20%	T, N, TNM	OS
Kim (2011) [25]	OC	90	IHC	NR	T, N	OS
Ryott (2011) [39]	OC	76	IHC	Score	TNM	None
Cha (2011) [26]	OC	103	IHC	Score	T, N, TNM	OS
Itoh (2003) [40]	OC	72	IHC	> 30%	T, N, TNM	OS, DFS
Chen (2013) [41]	LC	80	IHC	> 10%	T, N, TNM	OS
Wildeman (2009) [42]	LC	59	IHC	> 5%	None	RFS
Kourelis (2009) [43]	LC	91	IHC	> 10%	T, N	None
Dong (2007) [44]	LC	68	IHC	> 5%	T, N	None
Cho (2004) [45]	LC	119	IHC	Score	None	OS, RFS
Ranelletti (2001) [46]	LC	61	IHC	Score	T, N, TNM	OS, RFS
Xu (2013) [47]	NPC	148	IHC	Score	T, N	OS
Pan (2012) [23]	NPC	111	IHC	> 25%	None	OS, DFS, RFS, DMFS
Kim (2011) [48]	NPC	38	IHC	> 25%	None	OS
Huang (2010) [49]	NPC	170	IHC	Score	None	OS, RFS, DMFS
Kim (2010) [50]	NPC	69	IHC	> 25%	TNM	OS
Loong (2009) [27]	NPC	58	IHC	Score	T, N	OS
Fang (2006) [51]	NPC	20	IHC	Score	T, TNM	None
Chen (2005) [52]	NPC	37	IHC	Score	N	None
Tan (2004) [53]	NPC	81	IHC	Score	TNM	None
Sun (2011) [54]	HNC	83	IHC	NR	T, N, TNM	None
Saba (2009) [24]	HNC	38	IHC	Score	T, N	OS
Kyzas (2005) [55]	HNC	68	IHC	> 5%	N, TNM	None
Gallo (2002) [56]	HNC	52	IHC	> 20%	TNM	OS, DFS
Yang (2013) [57]	HPC	80	IHC	> 50%	None	OS
Sackett (2008) [58]	GC	301	IHC	> 50%	None	OS, RFS
Chang (2004) [59]	OPC	82	IHC	Score	T, N, TNM	OS, RFS

Abbreviations: OC, oral cancer; LC, laryngeal cancer; NPC, nasopharyngeal carcinoma; HNC, head and neck cancer; HPC, hypopharyngeal cancer; GC, glottic cancer; OPC, oropharyngeal cancer; Cox2, cyclooxygenase-2; IHC, immunohistochemistry; NR, not reported; T, tumor stage; N, lymph node metastasis; TNM, TNM stage; OS, overall survival; DFS, disease-free survival; RFS, recurrence-free survival; DMFS, distant metastasis-free survival.

## RESULTS

### Search results

**Figure 1:**
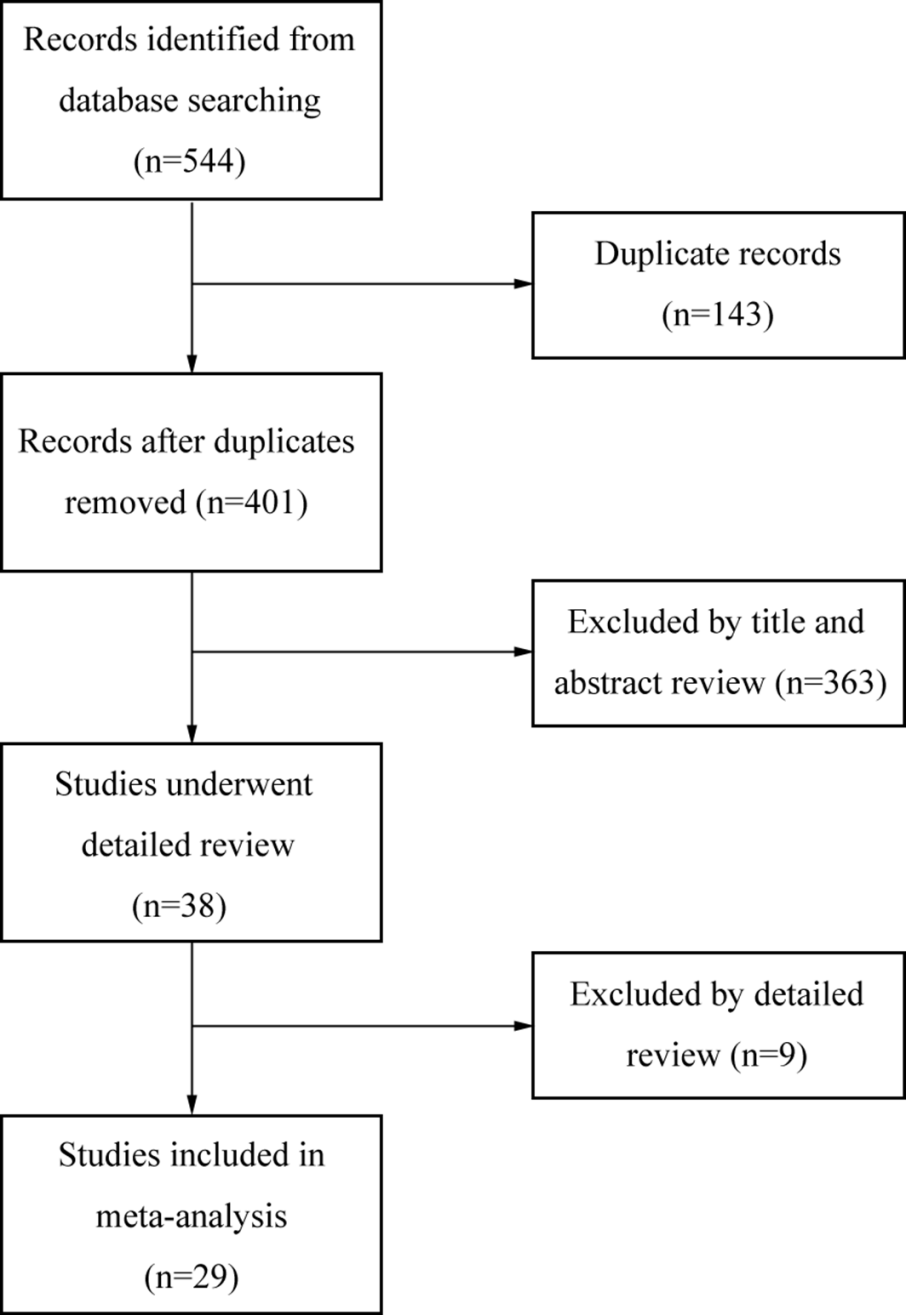
Flow diagram of study selection

Literature search and eligibility assessment were performed independently by 2 reviewers and disagreements among them were resolved by consensus. Figure 1 illustrated the process of study selection. 544 studies were initially found by our search strategy. After the article titles and abstracts were checked, 38 articles were reviewed in detail [22–59]. 9 studies were excluded as they did not provide sufficient data for extracting odds ratio (OR) or hazard ratio (HR) or 95% confidence intervals (CI) [28–36], leaving 29 studies that fulfilled the eligibility criteria [22–27, 37–59] (Table 1). Four studies reported no site-specific HNC [24, 54–57] and others recorded site-specific HNC. There were 7, 6 and 9 studies focused on oral cancer (OC) [22, 25, 27, 37–40], laryngeal cancer (LC) [41–46] and nasopharyngeal cancer (NPC) [23, 26, 47–53], respectively. The total number of patients was 2430, ranging from 20 to 301 cases per study. Immunohistochemistry (IHC) was the only technique used to detect the expression of COX-2. Twenty-two studies were dealing with clinicopathological factors, twenty were about survival results and thirteen studies evaluated both of them.

### Correlation of COX-2 expression with clinicopathological parameters

**Figure 2:**
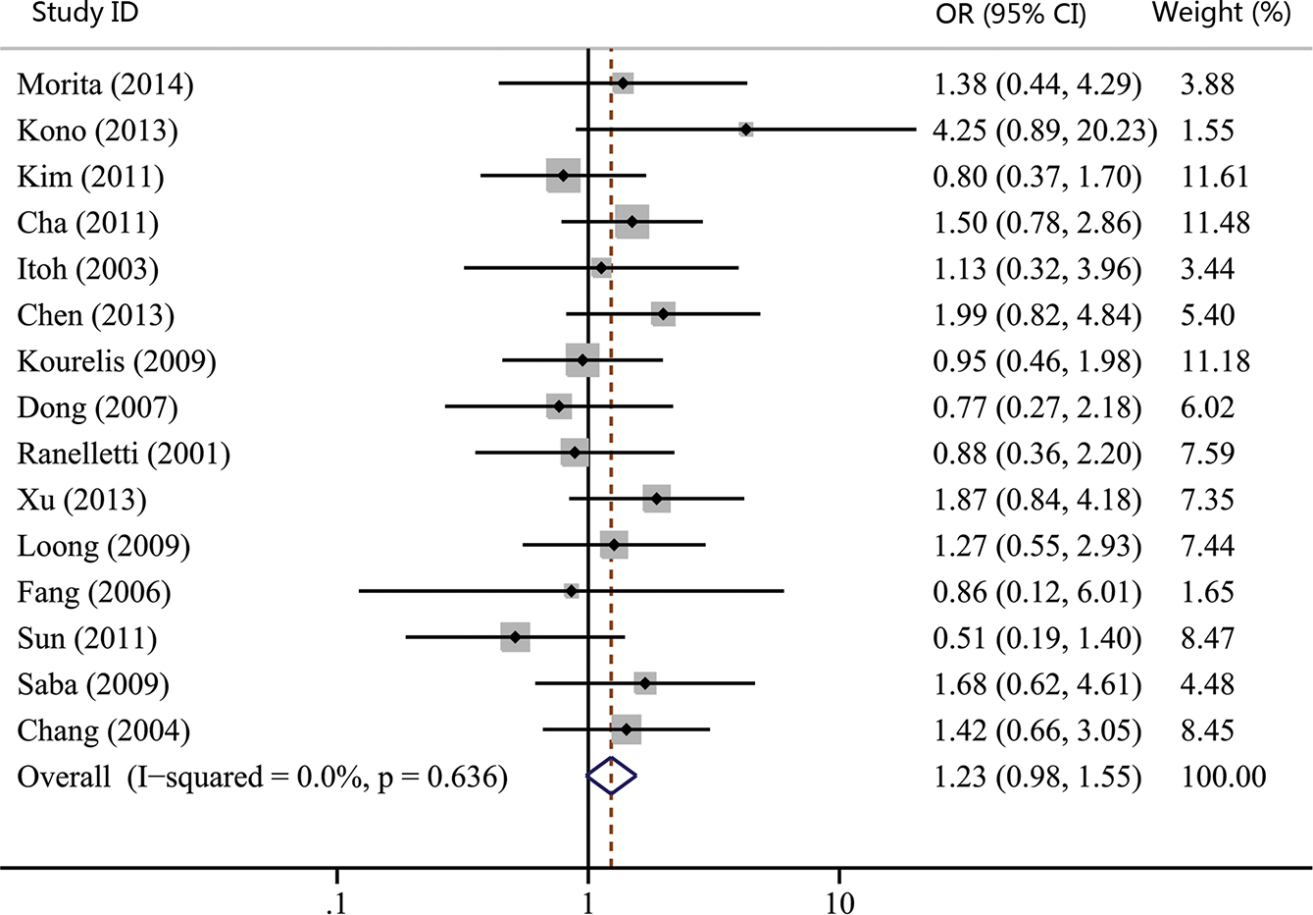
Forest plot of odds ratio (OR) for the association between COX-2 expression and advanced tumor stage in head and neck cancer CI, confidence interval.

**Figure 3:**
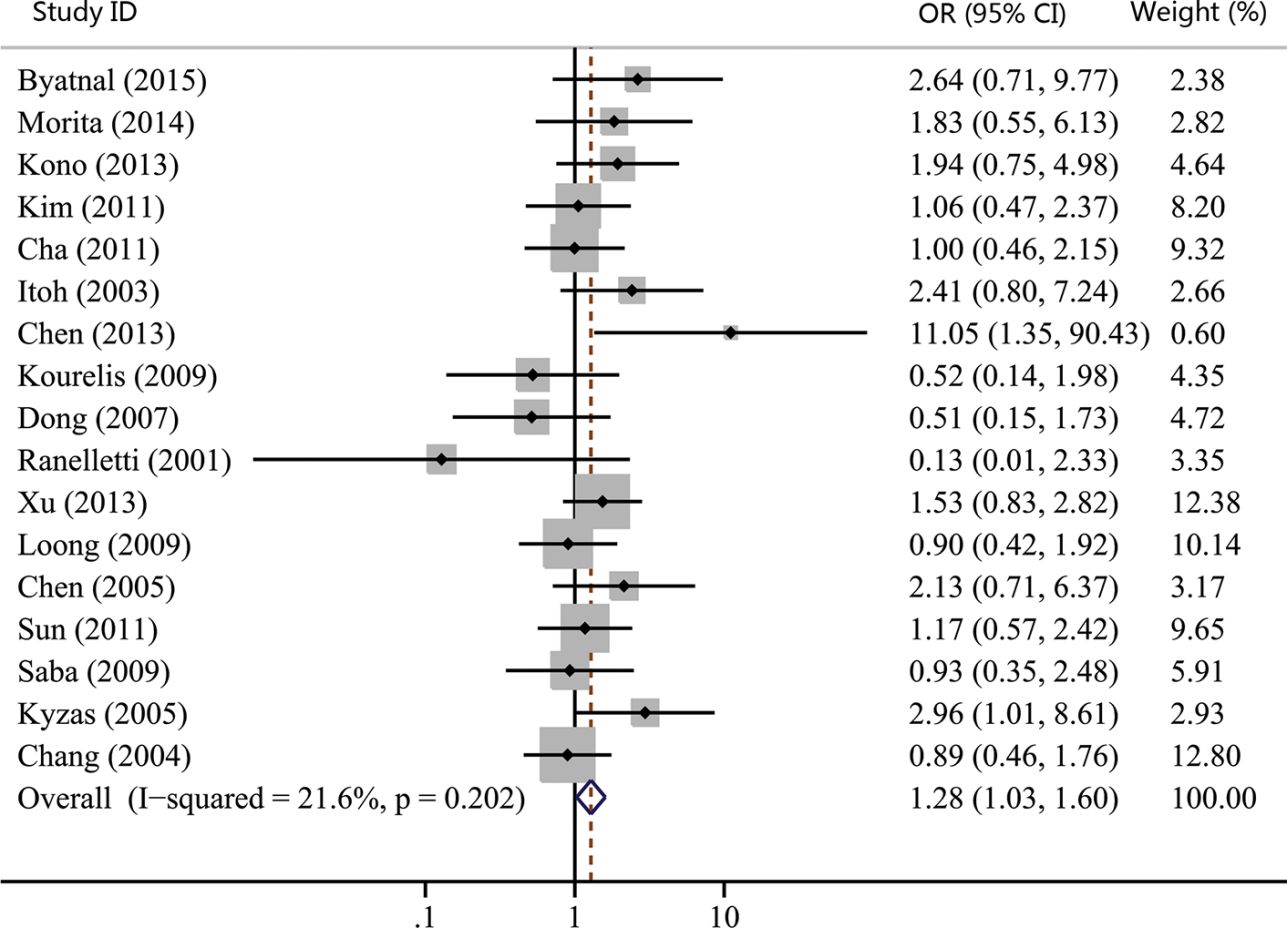
Forest plot of odds ratio (OR) for the association between COX-2 expression and lymph node metastasis in head and neck cancer CI, confidence interval.

**Figure 4:**
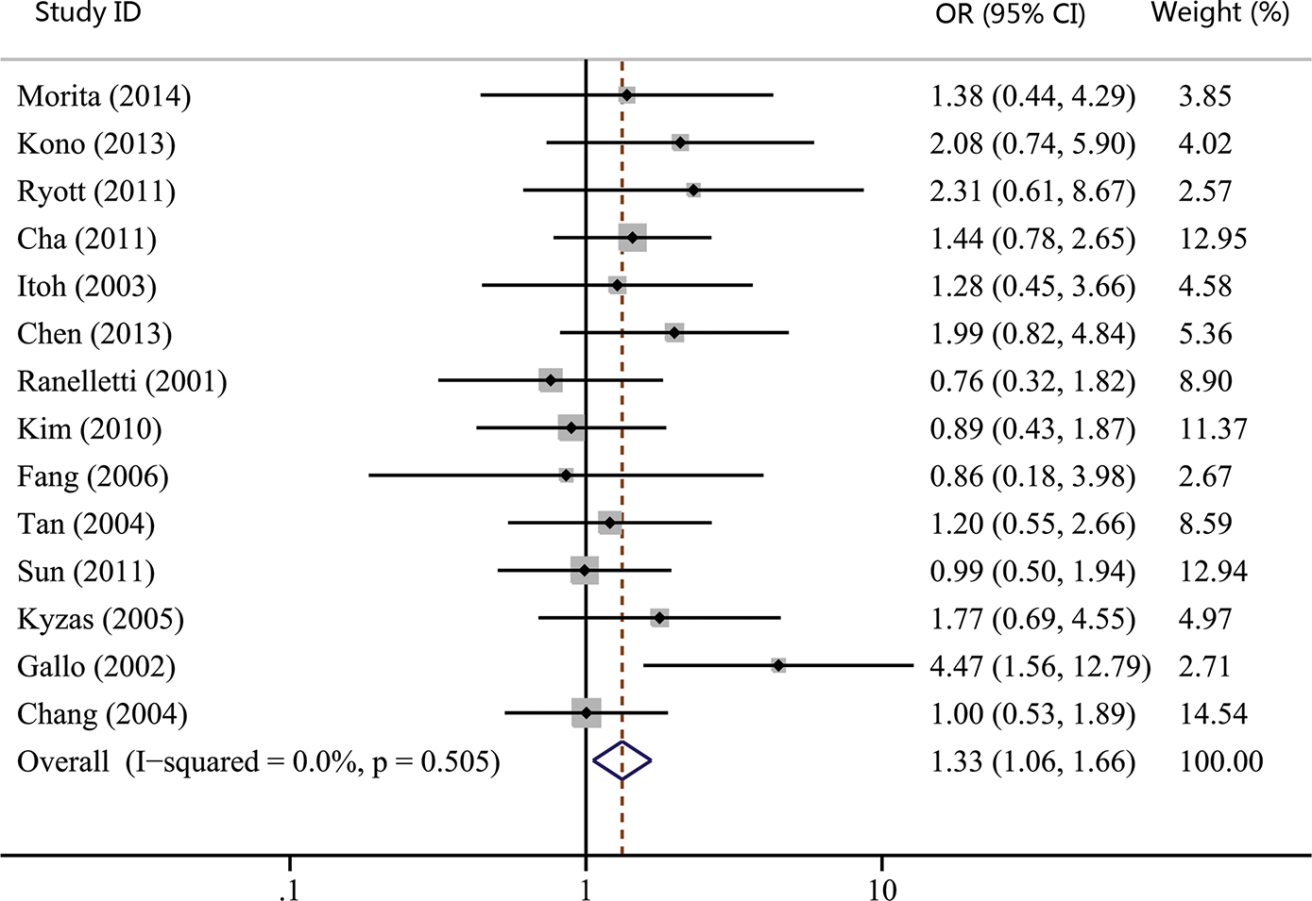
Forest plot of odds ratio (OR) for the association between COX-2 expression and advanced TNM stage in head and neck cancer CI, confidence interval.

The associations between COX-2 expression and advanced tumor stage, high risk of lymph nodal metastasis, and advanced TNM stage were reported by 15, 17, and 14 studies, respectively. Our results showed that COX-2 expression was not significantly correlated with advanced tumor stage (OR, 1.23; 95% CI, 0.98–1.55; *p* = 0.074; Figure 2). However, statistical significance between COX-2 expression and high risk of lymph node metastasis (OR, 1.28; 95% CI, 1.03–1.60; *p* = 0.027; Figure 3) and advanced TNM stage were found (OR, 1.33; 95% CI, 1.06–1.66; *p* = 0.015; Figure 4). Moreover, no or slight heterogeneity was observed in these analyses (*I*^2^ = 0%, *p* = 0.636 for tumor stage; *I*^2^ = 21.6%, *p* = 0.202 for lymph node metastasis; *I*^2^ = 0%, *p* = 0.505 for TNM stage).

Subgroup analyses were performed to examine the correlation of COX-2 expression with clinicopathological parameters in different tumor subtypes (Table 2). For tumor stage, the combined OR of subgroup analyses were displayed as follows: OC group (OR, 1.32; 95% CI, 0.89–1.97; *p* = 0.173); LC group (OR, 1.08; 95% CI, 0.70–1.67; *p* = 0.721); NPC group (OR, 1.50; 95% CI, 0.86–2.60; *p* = 0.150); and no site-specific HNC group (OR, 0.92; 95% CI, 0.46–1.83; p = 0.808). These results again indicated that COX-2 expression was not correlated with advanced tumor stage. Subgroup analyses suggested a positive correlation between COX-2 expression and high possibility of lymph node metastasis in patients with OC (OR, 1.49; 95% CI, 1.01–2.20; *p* = 0.043). But we failed to reveal a statistically significant association in patients with LC (OR, 0.90; 95% CI, 0.46–1.76; *p* = 0.763), NPC (OR, 1.36; 95% CI, 0.88–2.09; *p* = 0.165) and no site-specific HNC (OR, 1.38; 95% CI, 0.83–2.28; *p* = 0.215). For TNM stage, the results of subgroup analyses showed that COX-2 expression was significantly correlated with advanced TNM stage in patients with OC (OR, 1.58; 95% CI, 1.05–2.37; *p* = 0.030) and no site-specific HNC (OR, 1.64; 95% CI, 1.02–2.62; *p* = 0.041). However, this correlation was not found in LC group (OR, 1.22; 95% CI, 0.67–2.25; *p* = 0.519) and NPC group (OR, 1.01; 95% CI, 0.61–1.67; *p* = 0.979).

**Table 2:** Subgroup results of meta-analysis and heterogeneity test

Subgroup	No. of studies	ES (95% CI)	*P* value	Heterogeneity test	
				*I*^2^(%)	*P* value
T, ES was described by OR					
All	15	1.23 (0.98–1.55)	0.074	0	0.636
OC	5	1.32 (0.89–1.97)	0.173	1.9	0.396
LC	4	1.08 (0.70–1.67)	0.721	0	0.469
NPC	3	1.50 (0.86–2.60)	0.150	0	0.682
HNC	2	0.92 (0.46–1.83)	0.808	62.9	0.101
N, ES was described by OR					
All	17	1.28 (1.03–1.60)	0.027	21.6	0.202
OC	6	1.49 (1.01–2.20)	0.043	0	0.604
LC	4	0.90 (0.46–1.76)	0.763	65.4	0.034
NPC	3	1.36 (0.88–2.09)	0.165	0	0.381
HNC	3	1.38 (0.83–2.28)	0.215	27.9	0.250
TNM, ES was described by OR					
All	14	1.33 (1.06–1.66)	0.015	0	0.505
OC	5	1.58 (1.05–2.37)	0.030	0	0.926
LC	2	1.22 (0.67–2.25)	0.519	56.4	0.130
NPC	3	1.01 (0.61–1.67)	0.979	0	0.843
HNC	3	1.64 (1.02–2.62)	0.041	64.8	0.058
OS, ES was described by HR					
All	19	1.93 (1.29–2.90)	0.001	87.9	0.000
OC	5	1.65 (0.90–3.03)	0.106	53.3	0.073
LC	3	4.80 (0.73–31.6)	0.103	90.6	0.000
NPC	6	1.51 (0.52–4.42)	0.452	94.5	0.000
HNC	2	1.79 (0.85–3.77)	0.128	55.6	0.013
RFS, ES was described by HR					
All	7	2.02 (1.00–4.08)	0.050	82.5	0.000
LC	3	2.35 (0.98–5.63)	0.055	49.3	0.139
NPC	2	2.24 (0.31–16.2)	0.422	94.3	0.000
DFS, ES was described by HR					
All	3	5.14 (2.84–9.31)	0.000	61.6	0.074

Abbreviations: ES, effect size; CI, confidence interval; OR, odds ratio; HR, hazard ratio; OC, oral cancer; LC, laryngeal cancer; NPC, nasopharyngeal carcinoma; HNC, head and neck cancer; T, tumor stage; N, lymph node metastasis; TNM, TNM stage; OS, overall survival; RFS, recurrence-free survival; DFS, disease-free survival.

### Impact of COX-2 expression on survival of HNC

**Figure 5:**
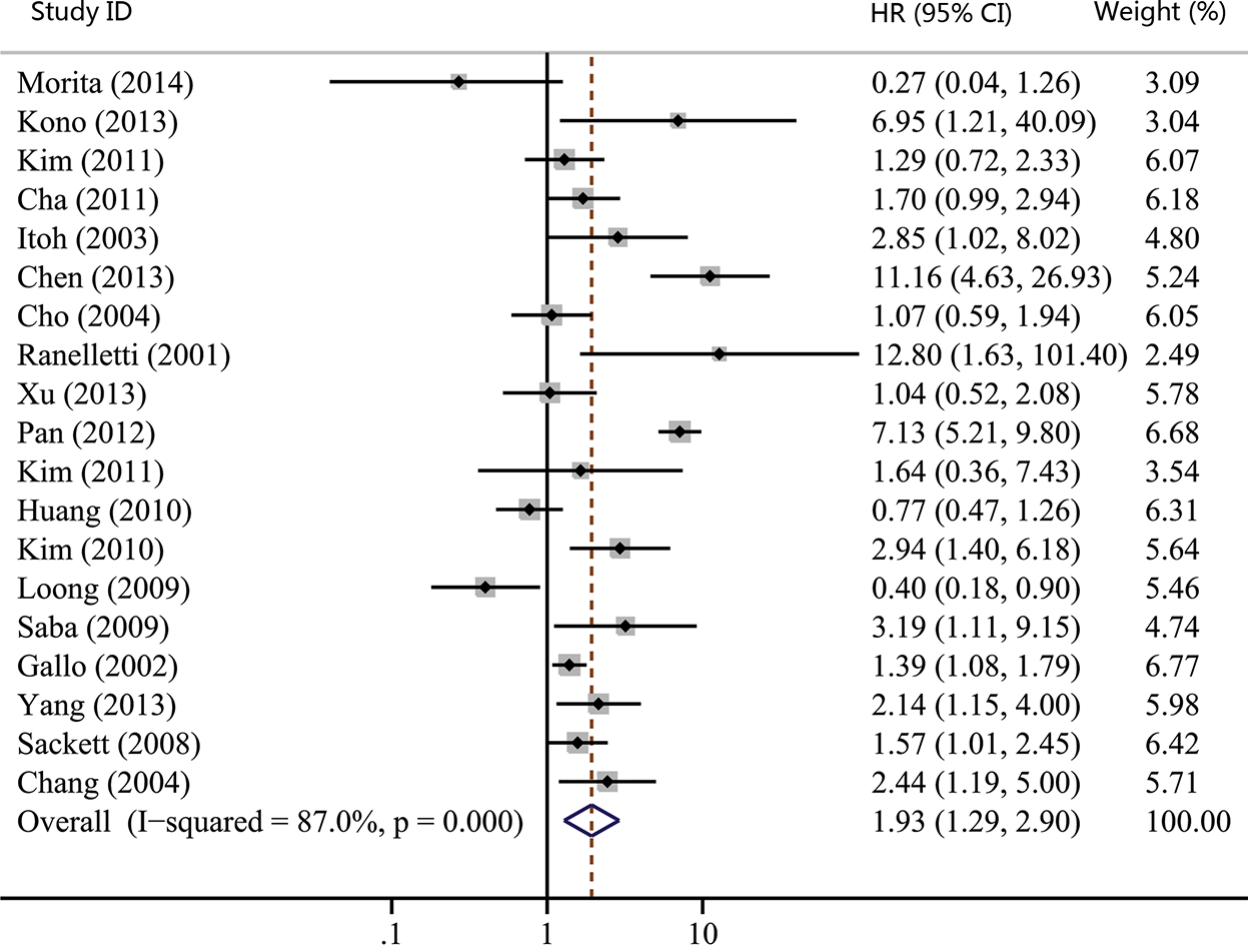
Forest plot of hazard ratio (HR) for the association between COX-2 expression and overall survival (OS) in head and neck cancer CI, confidence interval.

**Figure 6:**
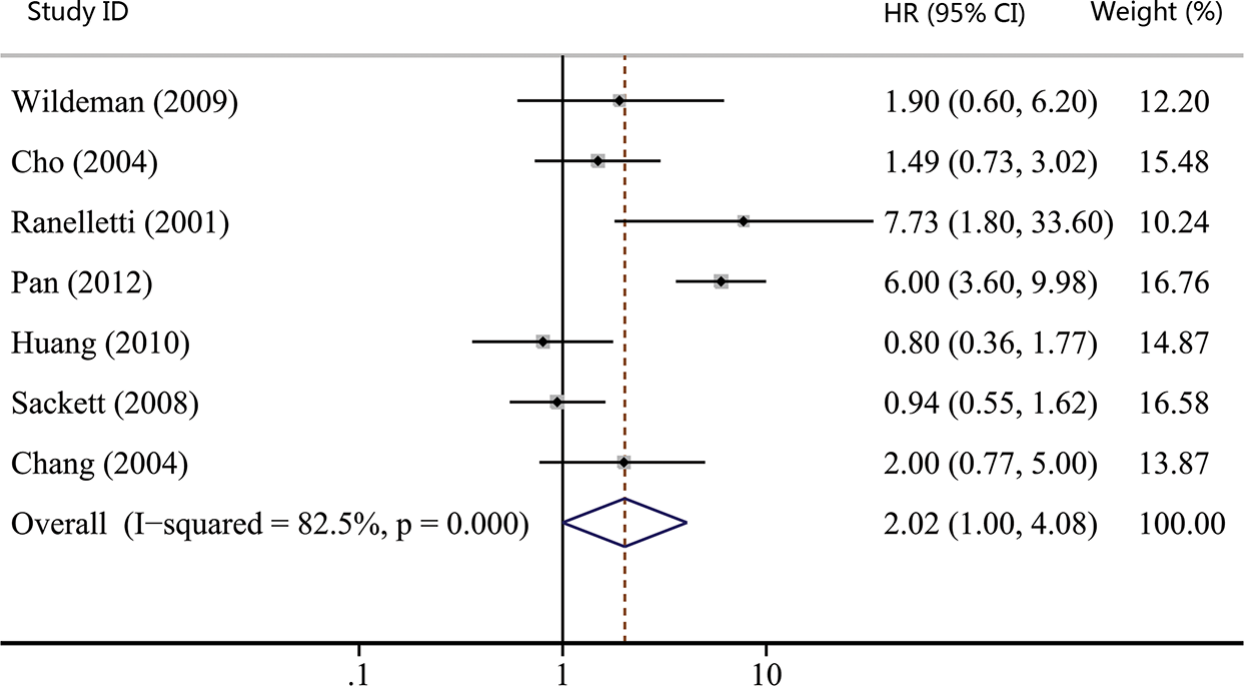
Forest plot of hazard ratio (HR) for the association between COX-2 expression and recurrence-free survival (RFS) in head and neck cancer CI, confidence interval.

**Figure 7:**
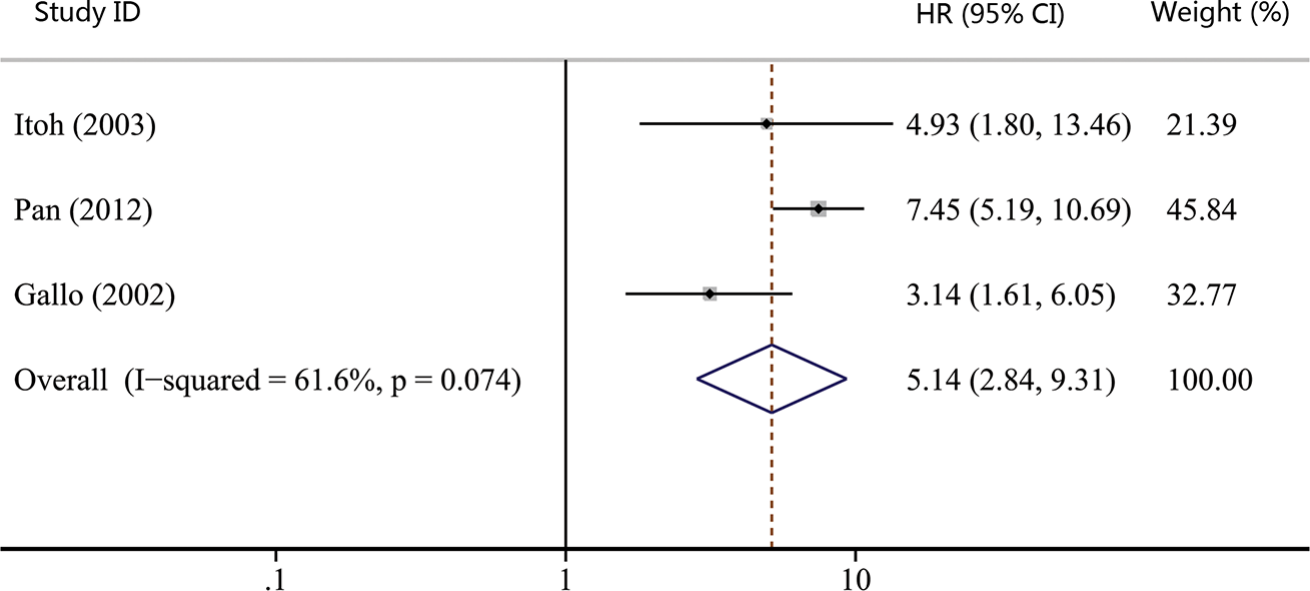
Forest plot of hazard ratio (HR) for the association between COX-2 expression and disease-free survival (DFS) in head and neck cancer CI, confidence interval.

As shown in Table 1, the number of studies reporting overall survival (OS), recurrence-free survival (RFS), disease-free survival (DFS) and distant metastasis-free survival (DMFS) were 19, 7, 3 and 2, respectively. Thus, we used OS, RFS and DFS as clinical outcomes in this meta-analysis. The combined HR for OS was 1.93 (95% CI, 1.29–2.90; *p* = 0.001), indicating that COX-2 expression had a significantly poor survival effect on patients with HNC (Figure 5). Similar to the results of OS, our study revealed that COX-2 expression was a poor predictor for RFS (HR, 2.02; 95% CI, 1.00–4.08; *p* = 0.050; Figure 6). Moreover, COX-2 expression indicated a low disease-free survival rate with a pooled HR of 5.14 (95% CI, 2.84–9.31; *p* = 0.000; Figure 7). The heterogeneity test showed a significant heterogeneity in these analyses (*I*^2^ = 87.9%, *p* = 0.000 for OS; *I*^2^ = 82.5%, *p* = 0.000 for RFS; *I*^2^ = 61.6%, *p* = 0.074 for DFS).

We also performed the subgroup analyses to evaluate the prognostic role of COX-2 in patients with site-specific cancers. The combined effect sizes were displayed in Table 2: OC group (HR, 1.65; 95% CI, 0.90–3.03; *p* = 0.106 for OS); LC group (HR, 4.80; 95% CI, 0.73–31.60; *p* = 0.103 for OS; HR, 2.35; 95% CI, 0.98–5.63; *p* = 0.055 for RFS); NPC group (HR, 1.51; 95% CI, 0.52–4.42; *p* = 0.452 for OS; HR, 2.24; 95% CI, 0.31–16.2; *p* = 0.422 for RFS); and no site-specific HNC group (HR, 1.79; 95% CI, 0.85–3.77; *p* = 0.128 for OS). Despite none of subgroup analyses showed statistically significant association between COX-2 expression and patients' survival, COX-2 expression had a tendency to suggest poor survival of HNC patients as the pooled HRs of all subgroup analyses were greater than 1.

### Publication bias

**Figure 8:**
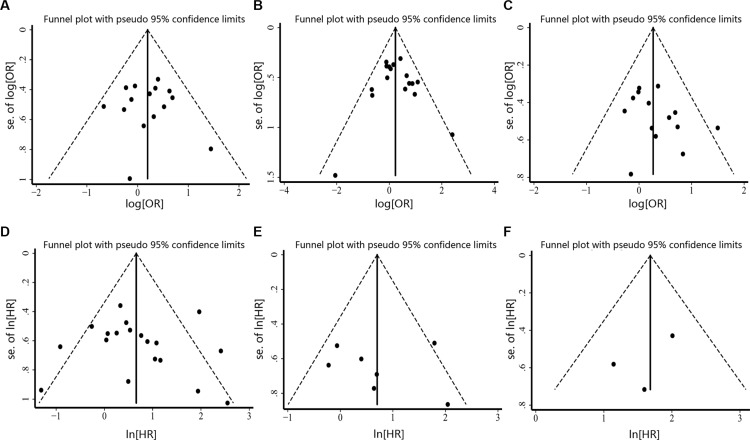
Funnel plot for the assessment of publication bias in this study (**A**) Funnel plot for 15 studies reporting tumor stage. (**B**) Funnel plot for 17 studies reporting lymph node metastasis. (**C**) Funnel plot for 14 studies reporting TNM stage. (**D**) Funnel plot for 19 studies reporting OS. (**E**) Funnel plot for 7 studies reporting RFS. (**F**) Funnel plot for 3 studies reporting DFS.

We assessed the publication bias by visually assessing a funnel plot for asymmetry and by quantitatively performing Begg's test and Egger's test. As shown in Figure 8, there was no clear evidence of funnel plot asymmetry by visual assessment. Moreover, publication bias statistics displayed in Table 3 indicated that no publication bias was detected either from Begg's test or Egger's test.

**Table 3:** Results of Begg's test and Egger's test for publication bias

Analysis	Studies	Begg's test (*p* value)	Egger's test (*p* value)
Tumor stage	15	1.000	0.879
Lymph node metastasis	17	0.711	0.602
TNM stage	14	0.274	0.162
Overall survival	19	0.108	0.910
Recurrence-free survival	7	0.548	0.955
Disease-free survival	3	1.000	0.487

## DISCUSSION

The prognostic value of COX-2 expression has been investigated extensively in various cancers [23–25]. These studies suggested that COX-2 expression could predict poor survival of cancer patients. In HNC, the association between COX-2 expression and clinical outcome has also been widely studied. However, it is still difficult to confirm the prognostic value of COX-2 expression in HNC as the existing studies are often small and conflicting in their results. In this study, we provided the evidence that COX-2 expression was significantly associated with high risk of lymph node metastasis and advanced TNM stage. In addition, our study indicated that COX-2 expression could act as an available prognostic factor for OS, RFS and DFS in patients with HNC.

To the best of our knowledge, this is the first comprehensive meta-analysis to confirm the clinicopathological value of COX-2 expression in HNC. The combined OR for COX-2 expression on high possibility of lymph node metastasis and advanced TNM stage were 1.28 (95% CI 1.03–1.60; *p* = 0.027) and 1.33 (95% CI 1.06–1.66; *p* = 0.015), suggesting that COX-2 expression was associated with tumor progression and metastasis. This is consistent with previous *in vitro* studies which showed that COX-2 was able to promote the proliferation, migration and invasion of HNC cells [60, 61]. Recently, Morita and his colleagues developed an animal model of oral squamous cell carcinoma (OSCC) to monitor the progression of lymph node metastases [62]. His results indicated that COX-2 was critical for the development of lymphatic metastasis in OSCC. On the other hand, Hu's serial studies have investigated the anticancer effect of celecoxib (a selective COX-2 inhibitor) on NPC cell lines [63, 64]. They found that celecoxib could inhibit the proliferation and invasion activity of various NPC cell lines.

Twenty studies were enrolled to investigate the effect of COX-2 expression on survival of HNC. We got the combined HR value of 1.93 (95% CI: 1.29–2.90; *p* = 0.001) for OS, which suggested that patients with COX-2 expression had a shorter overall survival time. In addition, the pooled HR for RFS was statistically significant (HR, 2.02; 95% CI: 1.00–4.08; *p* = 0.050), indicating that COX-2 expression is a predictor of worse recurrence-free survival. A significant association was also found between COX-2 expression and poor DFS (HR, 5.14; 95% CI, 2.84–9.31; *p* = 0.000). These results hinted that COX-2 expression could work as a prognostic factor for patients with HNC. Radiotherapy and platinum-based chemotherapy are important treatment options for HNC [65, 66]. Several reports have revealed that COX-2 relates with radiosensitivity and platinum resistance of HNC [67, 68], which testifies the prognostic role of COX-2 on the other side. Moreover, the effect of COX-2 inhibitors has been analyzed clinically for patients with HNC. Prof. Prabhash proposed a metronomic chemotherapy (MCT) consisting of celecoxib and methotrexate [20] and conducted a prospective study comparing MCT with intravenous cisplatin (IP) in patients with metastatic, relapsed or inoperable squamous cell carcinoma of head and neck [21]. Patients in the MCT arm had significantly longer progression-free survival (PFS) and OS compared to the IP arm (median PFS: 101 vs. 66 days; *p* = 0.014, median OS: 249 vs. 152 days; *p* = 0.02). His studies suggested that inexpensive COX-2 inhibitor might be a good option for palliative chemotherapy in patients with HNC, especially for patients in lesser developed countries. Thus, COX-2 expression correlates with poor survival and targeting COX-2 may be an effective way to control HNC.

As head and neck cancer is a heterogeneous disease with various cancer types, we also conducted subgroup analyses in this study. However, the results of subgroup analyses were not always consistent with that of pooled analyses. For tumor stage, subgroup analyses indicated that COX-2 expression was not correlated with advanced tumor stage, which was in accord with the whole analysis. But for lymph node metastasis and TNM stage, subgroup analyses only revealed that COX-2 expression was statistically related with high possibility of lymph node metastasis in OC and advanced TNM stage in OC and no site-specific HNC. Moreover, subgroup analyses only showed a tendency without statistically significant association between COX-2 expression and survival. Two critical factors may explain this situation. First, the etiologies of HNC vary in different tumor subtypes. Human papillomavirus (HPV) infection is a well accepted risk factor for the development of OC [69, 70], while Epstein-Barr virus (EBV) infection is closely associated with NPC [71]. The effect of COX-2 in site-specific cancers may differ with each other. Second, the limited number of articles adopted in subgroup analyses may make the pooled effect sizes differ from their true value. As showed in Table 2, the number of studies included in per subgroup analysis ranged from 2 to 6 and the majority of them were 3. The limited studies for subgroup analyses may affect the real results of subgroup analyses. Considering these points, future large-scaled studies are still needed to improve our results.

Estimating heterogeneity and publication bias is an essential part of a meta-analysis. In this study, heterogeneity test revealed no significant heterogeneity when analyzing the associations between COX-2 expression and advanced tumor stage, high risk of lymph node metastasis and advanced TNM stage. But heterogeneity appeared when assessing the prognostic value of COX-2 expression. The heterogeneity may partly come from the variations in assessing COX-2 expression. Although IHC was the only method used to detect COX-2 expression in these studies, large variability was presented when defining COX-2 overexpression. First, some studies defined COX-2 status based on the staining extents, whereas others used a scoring system. Second, the cutoff values for judging COX-2 overexpression varied with studies, ranging from 5% to 50% when the staining extents were used as cut-off points. The standardization of COX-2 overexpression assay may resolve this problem in the future. With regard to publication bias, no clear evidence of funnel plot asymmetry was found by visual assessment. Moreover, no publication bias was detected according to both Begg's test and Egger's test. These findings suggested that our results were robust and not far from the actual situation.

However, the present meta-analysis still has several limitations. First, this study is a literature-based meta-analysis, making our results less reliable than individual patient data-based analysis. Second, significant heterogeneity was noted when analyzing the association between COX-2 expression and patients' survival. Third, studies that cannot provide sufficient data to extract OR or HR were excluded. The exclusion of these studies may make our pooled effect sizes differ from their true value on some level.

In view of this study, our findings showed that COX-2 expression correlated with high risk of lymph node metastasis and advanced TNM stage in HNC. Moreover, COX-2 expression indicated poor OS, RFS and DFS in patients with HNC. In conclusion, COX-2 expression can act as a prognostic factor for HNC, which might help to define high risk patients and guide clinical decision making. However, there are two important questions that need to be further answered. First, significant heterogeneity was noted when analyzing the impact of COX-2 expression on survival. Second, the results of subgroup analyses were not always consistent with that of pooled analyses. Only a tendency without statistically significant association between COX-2 expression and patients' survival was showed in subgroup analyses. Considering these issues, our results need to be validated and updated in the near future.

## MATERIALS AND METHODS

### Literature search

Electronic searches for relevant articles in PubMed, Embase, and Web of Science databases were conducted in December 2015. The search strategy was generated by combining key words related to COX-2 (‘cyclooxygenase-2′ or ‘COX-2′), HNC (‘head and neck cancer’ or ‘oral carcinoma’ or ‘oral cancer’ or ‘pharyngeal carcinoma’ or ‘pharyngeal cancer’ or ‘laryngeal carcinoma’ or ‘laryngeal cancer’ or ‘nasopharyngeal cancer’ or ‘nasopharyngeal carcinoma’), and prognosis (‘prognosis’ or ‘prognostic’). Moreover, we manually searched the reference lists of relevant articles for additional publications.

### Inclusion criteria

Studies were included in this meta-analysis if they met the following criteria: a) all patients recruited in the study were diagnosed with HNC; b) COX-2 expression was evaluated in primary tumor tissues; c) the clinicopathological or prognostic value of COX-2 expression was tested in the article; d) only English-language studies were included; e) the OR or HR and their corresponding 95% CIs were described or could be statistically extracted from the study; f) When several articles were from the same patient population, the newest or most informative single article was included.

### Information extraction

The following information was extracted from each study: first author's last name, publication year, tumor types, number of patients, COX-2 expression assay (method and cut-off level), clinicopathological data (number of patients with different tumor stage, lymph node stage, and TNM stage), and survival data (HR and its 95% CIs for OS, RFS, and DFS).

### Statistical analysis

According to clinical characteristics, T3 and T4 were combined as advanced tumor stage; TNM stage III and stage IV were combined as advanced TNM stage. OR and its 95% CIs were used to describe the correlation between clinicopathological factors and COX-2 status. HR and its 95% CIs were adopted to estimate the prognostic value of COX-2 expression. The individual OR or HR estimates were combined into an overall OR or HR and the results were presented graphically in the form of a forest plot. Pooled effect sizes were considered to be significantly different if their 95% CIs did not include 1 (*p* < 0.05). OR > 1 or HR > 1 implied a poor survival for the COX-2 expression group. The Cochran *Q* test and *I*^2^ test were performed to assess the heterogeneity between studies. When the Cochran *Q* test *p* value was ≤ 0.10 and *I*^2^ test *I*^2^ value was ≥ 50%, statistically significant heterogeneity was considered to be present. When heterogeneity was absent, fixed effects models were employed; otherwise, random effects models were adopted. Funnel plots, Begg's test, and Egger's test were performed to test publication bias. All analyses were carried out by using Stata Statistical Software, version 12.0 (Stata Corporation, College Station, TX, USA).
